# Effect of Proxy Responses on Tobacco Use Surveys in Thailand, 2011

**DOI:** 10.5888/pcd15.180158

**Published:** 2018-10-25

**Authors:** Jason Hsia, Hataichanok Puckcharern, Machell Town

**Affiliations:** 1 Division of Population Health, Centers for Disease Control and Prevention, Atlanta, Georgia; 2 Division of Statistical System Management, National Statistical Office, Bangkok, Thailand

## Abstract

Proxy responses are often allowed in household tobacco surveys when all household members are included in a sample. To assess the effect of proxy responses on prevalence estimates, we compared 2 surveys in 2011 that gauged tobacco use in Thailand: the Cigarette Smoking and Alcohol Drinking Survey (SADS) and the Global Adult Tobacco Survey (GATS). Both surveys had similar nonsampling errors and design, but SADS allowed proxy responses and GATS did not. When proxy responses were included in SADS, the prevalence estimate was 10% lower in GATS for men (41.69% in GATS vs 46.55% in SADS) and 18% lower in GATS for women (2.14% in GATS vs 2.61% in SADS). Eliminating proxy responses is recommended to increase accuracy of tobacco-use surveillance.

## Objective

Household surveys with equal selection probability design often use selection probability proportional to size in the first few stages and simple random sampling to select a fixed household number in the last stage; then they interview all members of selected households. Although this design may have smaller sampling errors than unequal probability selection designs have, proxy responses introduce nonsampling errors, because of the varying availability of household members for interviews. The objective of this study was to assess the effect of proxy responses on estimating the prevalence of tobacco use in Thailand.

## Methods

We used data from 2 tobacco-use surveys in Thailand that differed in proxy use but had similar sample design and other nonsampling errors (Appendix). The Smoking and Alcohol Drinking Survey (SADS) has been used for surveillance of tobacco and alcohol use in Thailand since 1991 (1). The Global Adult Tobacco Survey (GATS) was first used for tobacco surveillance in 2009 (2). SADS and GATS target noninstitutionalized persons (aged ≥11 in SADS and ≥15 in GATS). Both surveys categorize geography into 5 regions (Bangkok, Central, North, Northeast, or South) and 2 levels of urbanicity (urban or rural). All primary sampling units (PSUs) were enumeration areas designated by the National Statistical Office of Thailand (1,2). The number of PSUs was proportionally allocated to each stratum of geography and urbanicity. For first-stage SADS, 4,830 PSUs were selected by using selection probability proportional to the number of households. The second stage randomly chose an equal number of households from previously selected PSUs. All eligible people in the randomly selected household were interviewed; 1 present adult household member answered questions for unavailable members. In the first stage of GATS sampling, 1,088 PSUs were selected by using the same sampling method that SADS used. The second stage of sampling in GATS was also the same as in SADS. At the third stage, only 1 randomly selected member from all eligible household members was interviewed. The GATS survey protocol requires that at least 3 attempts are made to visit the selected household member; proxy responses are prohibited. Our comparison was based on 177,350 persons in SADS and 21,488 persons in GATS (all aged ≥15).

Thailand’s National Statistical Office conducted the surveys with similar interviewers, supervisors, interview techniques, response rates, and quality of fieldwork. Using the most recent survey data available (2011), we assessed a key survey variable, current smoking, for proxy responses. In Thailand, more than 95% of tobacco users are cigarette smokers; the prevalence of tobacco use is low among women. We calculated and compared current smoking prevalence and their 95% confidence intervals (CIs) for SADS (with and without proxy responses) and GATS.

## Results

The response rate was 97.2% for SADS and 97.7% for GATS at the household level and 98.5% for GATS at the individual level. The proxy rate was 42.6% for SADS. Of proxy responses, 81.7% were provided by participants aged 45 or older and 67.9% were provided by women.

The prevalence of current smoking decreased for both men and women from 1991 to 2004 and then stayed flat thereafter in SADS (Figure). Estimates in SADS were lower than estimates in GATS in 2009 and 2011. In a comparison of GATS and SADS in 2011, when proxy responses were included in SADS, the prevalence estimate was 10% lower in GATS for men (41.69% vs 46.55%) and 18% lower in GATS for women (2.14% vs 2.61% ) (Table). The percentage-point differences between SADS and GATS were −4.87 (95% CI, −6.60 to −3.13; *P* < .001) for men and −0.47 (95% CI, −0.93 to −0.02; *P* = .04) for women. When proxy responses in SADS were excluded, the estimated prevalence in SADS increased to 45.27% for men and 2.86% for women in 2011, not significantly different from the estimates in GATS. We found similar results by age, residence, region, and income for all subgroups of men and most subgroups of women (Table). We found a different pattern for some subgroups of women. For example, among women aged 15 to 24, the prevalence increased when we excluded proxy responses in SADS, but the difference between the estimate in SADS (1.33%) was significantly different from the estimate in GATS (0.43%). With few exceptions, the estimated prevalence was higher in SADS after excluding proxy responses, and these estimates were closer to GATS estimates for men and women overall and among subgroups.

**Figure:**
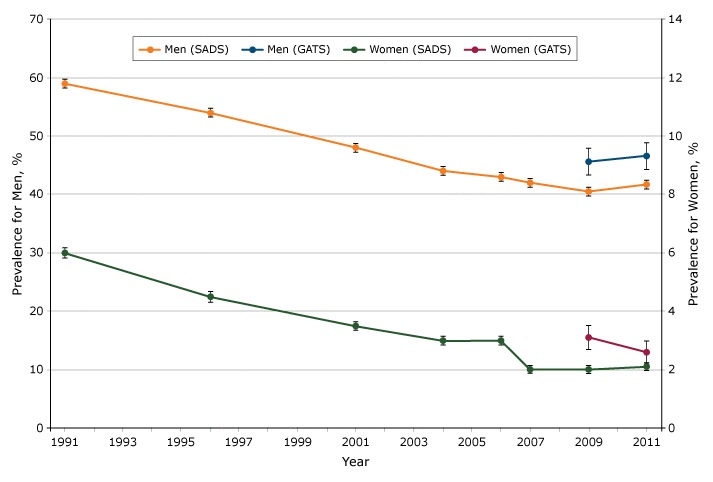
Prevalence of current smoking estimated from SADS and GATS in Thailand, 2011. No data were available from GATS from 1991 through 2007. Error bars indicate 95% confidence intervals. Abbreviations: GATS, Global Adults Tobacco Survey (2); SADS, Cigarette Smoking and Alcohol Drinking Survey (1). **Table:** 

Year	SADS		GATS	
	Men, % (95% Confidence Interval)	Women, % (95% Confidence Interval)	Men, % (95% Confidence Interval)	Women, % (95% Confidence Interval)
1991	59.0 (58.3–59.7)	6.0 (5.8–6.2)	—	—
1996	54.0 (53.3–54.7)	4.5 (4.3–4.7)	—	—
2001	48.0 (47.3–48.7)	3.5 (3.3–3.7)	—	—
2004	44.0 (43.3–44.7)	3.0 (2.9–3.1)	—	—
2006	43.0 (42.3–43.7)	3.0 (2.9–3.1)	—	—
2007	42.0 (41.3–42.7)	2.0 (1.9–2.1)	—	—
2009	40.5 (39.8–41.2)	2.0 (1.9–2.1)	45.6 (43.3–47.9)	3.1 (2.7–3.5)
2011	41.7 (41.0–42.4)	2.1 (2.0-2.2)	46.6 (44.3–48.9.3)	2.6 (2.2–3.0)

**Table:** Estimated Prevalence of Current Smoking From 2011 SADS, Including and Excluding Proxy Responses, and 2011 GATS, by Demographic Characteristics

Variable	SADS Including Proxy (*a*)	GATS (*b*)	SADS Excluding Proxy (*c*)	*a *− *b*, Percentage Point (95% CI) [*P* Value^a^]	*c *− *b*, Percentage Point (95% CI) [*P* Value^b^]
**Men**					
**All**	41.69	46.55	45.27	−4.87 (−6.60 to −3.13) [<.001]	−1.29 (−3.02 to 0.45) [.15]
**Age**					
15–24	31.76	42.00	42.76	−10.24 (−13.66 to −6.82) [<.001]	0.76 (−2.67 to 4.19) [.66]
25–44	47.59	50.50	49.74	−2.90 (−5.69 to −0.11) [.04]	−0.76 (−3.55 to 2.03) [.59]
45–59	45.11	48.74	46.73	−3.63 (−7.10 to −0.16) [.04]	−2.01 (−5.48 to 1.46) [.26]
≥60	32.61	38.30	34.43	−5.68 (−9.05 to −2.31) [.001]	−3.86 (−7.24 to −0.49) [.02]
**Residence**					
Urban	34.67	39.63	37.79	−4.96 (−7.36 to −2.56) [<.001]	−1.84 (−4.25 to 0.56) [.13]
Rural	45.23	50.08	49.36	−4.85 (−7.30 to −2.39) [<.001]	−0.72 (−3.17 to 1.74) [.57]
**Region**					
Bangkok	32.06	36.51	37.45	−4.45 (−8.18 to −0.72) [.02]	0.95 (−2.79 to 4.69) [.62]
Central	37.38	44.54	41.69	−7.16 (−11.02 to −3.31) [<.001]	−2.86 (−6.71 to 1.00) [.15
North	37.04	39.35	40.66	−2.32 (−5.34 to 0.71) [.13]	1.31 (−1.75 to 4.37) [.40]
Northeast	46.50	49.70	50.22	−3.20 (−6.33 to −0.07) [.045]	0.52 (−2.61 to 3.65) [.74]
South	50.52	59.24	53.66	−8.72 (−13.81 to −3.63) [.001]	−5.58 (−10.66 to −0.49) [.03]
**Income**					
Lowest third	39.77	47.83	45.56	−8.06 (−11.06 to −5.06) [<.001]	−2.27 (−5.28 to 0.73) [.14]
Middle third	48.94	51.54	50.72	−2.59 (−5.10 to −0.08) [.04]	−0.81 (−3.33 to 1.71) [.53]
Highest third	34.91	40.03	37.30	−5.12 (−8.07 to −2.18) [.001]	−2.73 (−5.68 to 0.21) [.07]

**Women**					
**All**	2.14	2.61	2.86	−0.47 (−0.93 to −0.02) [.04]	0.25 (−0.22 to 0.72) [.30]
**Age**					
15–24	0.76	0.43	1.33	0.33 (0.04 to 0.62) [.03]	0.90 (0.58 to 1.22) [<.001]
25–44	1.71	2.34	2.15	−0.63 (−1.26 to −0.01) [.05]	−0.19 (−0.84 to 0.46) [.56]
45–59	2.95	3.71	3.48	−0.76 (−1.47 to −0.05) [.06]	−0.23 (−1.04 to 0.58) [.58]
≥60	3.42	3.90	4.16	−0.48 (−0.94 to −0.02) [.25]	0.26 (−0.58 to 1.10) [.54]
**Residence**					
Urban	1.75	2.98	2.30	−1.22 (−1.88 to −0.57) [<.001]	−0.67 (−1.34 to 0.01) [.048]
Rural	2.35	2.41	3.16	−0.06 (−0.52 to 0.39) [.79]	0.75 (−0.07 to 1.23) [.002]
**Region**					
Bangkok	1.57	2.80	2.25	−1.23 (−1.99 to −0.48) [.001]	−0.55 (−1.33 to 0.24) [.17]
Central	2.24	3.69	3.06	−1.45 (−2.32 to −0.58) [.001]	−0.63 (−1.53 to 0.26) [.17]
North	4.68	4.71	5.90	−0.03 (−1.03 to 0.95) [.96]	1.19 (−0.06 to 2.21) [.02]
Northeast	1.08	1.04	1.50	0.04 (−0.44 to 0.52) [.88]	0.45 (−0.04 to 0.94) [.07]
South	1.54	1.50	1.89	0.04 (−0.53 to 0.62) [.89]	0.39 (−0.19 to 0.97) [.19]
**Income**					
Lowest third	2.32	3.10	3.18	−0.78 (−1.40 to −0.15) [.01]	0.07 (−0.57 to 0.72) [.82]
Middle third	2.22	2.65	2.82	−0.42 (−0.83 to −0.01) [.04]	0.17 (−0.42 to 0.76) [.58]
Highest third	1.24	1.75	1.74	−0.50 (−0.97 to −0.03) [.04]	−0.01 (−0.49 to 0.48) [.98]

Abbreviation: CI, confidence interval; GATS, Global Adults Tobacco Survey (2); SADS, Cigarette Smoking and Alcohol Drinking Survey (1).

a *z* Test used to determine whether estimated proportion from *a* is the same as from *b*.

b *z* Test used to determine whether estimated proportion from *c* is the same as from *b*.

## Discussion

SADS and GATS in Thailand in 2011 provided a rare opportunity to compare prevalence estimates. Whereas SADS used equal probability sample and proxy responses, GATS used unequal probability sample and no proxy responses. The same authoritative statistical agency conducted both surveys, using similar interviewers, supervisors, and interview techniques and following the same field-operation protocol. Nonsampling errors other than those due to proxy responses, therefore, can be assumed to be similar to each other or at least not a major cause of nonsampling error in the comparison. When proxy responses were removed and prevalence estimates in SADS were recalculated, the estimates increased and were much closer to those in GATS, indicating that proxy reports generate lower estimates of smoking rates than self-reported data. Previous studies reported similar findings (3,4).

This study has several limitations. First, the study was conducted in Thailand, and the extent to which stigma about smoking may affect proxy responses is not known. For example, some family members may wish to hide another family member’s smoking behavior. Some proxy validation studies in the United States did not find this effect (5,6); in the United States, family members usually do not hide the smoking behavior of other family members. Second, small numbers of female smokers might have resulted in unstable estimates among subgroups of women in GATS, these unstable estimates might have affected the subgroup analysis among women, such as those aged 15 to 24. Third, older household members possibly acted as proxies for the more-often unavailable younger members. In the past decade, Thailand has developed a greater social tolerance for younger female smokers (1), and thus, household proxies may have had less of an effect on the reporting of stigmatized behaviors than they did previously (3).

The China Health Service Survey had similar findings in its multistage cluster sampling design, which permitted household proxies. The prevalence of current smoking among men in the China Health Service Survey was lower (46.5%) than the prevalence in a survey that had a similar design but prohibited household proxies (52.1%) (7,8). In summary, the results of our research in Thailand suggest that proxy responses generally lead to an underestimation of the prevalence of current smokers.

## Appendix Survey Questions, Sample Design, and Survey Operation of 2011 Smoking and Alcohol Drinking Survey and 2011 Global Adults Tobacco Survey in Thailand

**Table:**

Survey Questions	2011 Smoking and Alcohol Drinking Survey	2011 Global Adults Tobacco Survey
**Current smoking**	Do you currently smoke tobacco on a daily basis, less than daily, or not at all?	Do you currently smoke tobacco on a daily basis, less than daily, or not at all? (daily, less than daily, not at all, don’t know)
Questions before current smoking question	Demographic questions	Demographic questions
Question after current smoking question	Have you smoked tobacco daily in the past? (yes, no, don’t know, refused)	Have you smoked tobacco daily in the past?(yes, no, don’t know, refused)
**Sample Design**	2-Stage stratified cluster sampling	3-Stage stratified cluster sampling
Strata	Bangkok and 4 regions by urbanicity	Bangkok and 4 regions by urbanicity
Primary sampling units	Enumeration areas defined by the National Statistical Office (NSO)	Enumeration areas defined by NSO
Sampling units at stage 2	Households	Households
Eligible persons	Persons aged ≥11 years living in primary residence	Persons aged ≥15 years living in primary residence
Sampling units at stage 3	Not applicable	Randomly selected 1 person from the previously selected household
**Survey operation**		
Field work dates	March–May 2011	September–November 2011
Interviewer composition	Designated NSO’s employees	Designated NSO’s employees
Interviewer training	Field interviewers/supervisors were divided into 15 training sessions. NSO provided trainings.	Field interviewers/supervisors were divided into 2 training sessions. NSO provided trainings.
Response rate at household level	97.2%	97.7%
Response rate at individual level	Not applicable	98.5%
Proxy rate	42.6%	Proxy was not allowed
**Weight computation**		
Base weight	Inverse of product of selection probability of 2-stage sampling	Inverse of product of selection probability of 3-stage sampling
Adjustment for nonresponse	Inverse response rate within primary sampling unit	Inverse response rate within primary sampling unit × adjustment factor derived from weighting class method by region, urbanicity, age, and sex
Poststratification adjustment	Weighting class method by region, urbanicity, age, and sex	Weighting class method by region, urbanicity, age, and sex
**Variance Estimation**	Linearization method using SAS	Linearization method using SAS
